# Strychniæ in Epilepsy

**Published:** 1869-07

**Authors:** 


STRYCHNIÆ IN EPILEPSY.—Walter Tyrrell, M. R. C. S., states that he has watched the effects of strychniæ upon various forms of epilepsy and has no hesitation in affirming that in a large majority of caees its effects are most beneficial. He found but three cases in which it produced no favorable result, and no case in which it produced an unfavorable effect. He gives a medium quantity as a dose, for a lengthened period, rather than carrying the dose very high at first. The best results are obtained from gr. $\frac{1}{10}$ to gr. $\frac{1}{8}$, twice a day, in solution, the system appearing to regain its nervous strength under the continued use of the medicine.—*Medical and Surgical Reporter.*

